# The perceptions of anatomy teachers for different majors during the COVID-19 pandemic: a national Chinese survey

**DOI:** 10.1080/10872981.2021.1897267

**Published:** 2021-03-15

**Authors:** Yu Yan, Xin Cheng, Changman Zhou, Xuesong Yang, Yun-Qing Li

**Keywords:** Anatomy education online, active learning, assessment, evaluation, clinical and nonclinical medicine, COVID-19 pandemic

## Abstract

During the spring semester of 2020, medical school anatomists in China were forced by the COVID-19 pandemic to transition from face-to-face educators or part-time online educators to full-time online educators. This nationwide survey was conducted to assess online anatomy education during the pandemic for medical students from nonclinical medicine and clinical medicine majors at medical schools in China via WeChat. The total of 356 responders included 293 responders from clinical medicine and 63 respondents from nonclinical medicine majors (i.e., 21 from preventive medicine, 13 from stomatology, and 29 from traditional Chinese medicine). The survey results showed that several aspects of online anatomy education were quite similar in clinical and nonclinical majors’ classes, including theoretical and practical sessions, active learning, assessments and evaluations. However, there were statistically significant differences in class size, implementation of active learning activities prior to the pandemic, and the evaluation of the effectiveness of online learning during the pandemic, between clinical and nonclinical medicine majors. These results indicated that, compared with teachers of anatomy courses in clinical medicine, teachers of nonclinical medicine majors using online learning in medical schools in China had relatively poor preparation for online learning in response to the unforeseen pandemic.

## Introduction

In early 2020, the unforeseen COVID-19 pandemic appeared abruptly and spread rapidly across the world, resulting in millions of people being infected globally by the novel coronavirus, many thousands of whom died [1–5]. On 31 December 2019, a small cluster of pneumonia cases with an unidentified aetiology were initially reported to have been found in Wuhan city of Hubei Province, China; subsequently, a novel coronavirus was identified andcalled severe acute respiratory syndrome coronavirus 2 (SARS-CoV-2) [5,6]. The coronavirus was subsequently identified in several other Asian countries in mid-January. On 23 January 2020, to control the spread of the new coronavirus, the Chinese government executed a modern form of quarantine: all transit in and out of Wuhan was shut down [7], and the lockdown policy was soon thereafter expanded to other cities in Hubei Province [8]. On 11 March 2020, the World Health Organization (WHO) officially declared the novel coronavirus outbreak a global pandemic [9]. As a result, almost every country has implemented restrictive measures involving a major population shift indoors and banning social interactions to help reduce the spread of COVID-19. Thus, the normal order of society and the way people live and work underwent a complete upheaval [10]. By the middle of March 2020, more than one hundred countries throughout the world shut down their schools, including medical schools of higher education [11,12]. Traditional face-to-face learning and other academic activities in university education were temporarily suspended worldwide, and teaching activities were moved to online coursework [13]. China was the first country to initiate a quarantine during the pandemic, and most medical schools in China have been offering online education to medical students for the entire spring semester.

Anatomy is a prerequisite for medical students and an essential course that enables students to excel in other basic medical courses. The contents of anatomy include systematic anatomy, regional anatomy, neuroanatomy, histology and embryology. Regarding the medical education system in China, the Soviet model of autonomous medical universities has been employed, in which higher medical education in China commonly offers 5- or 6-year medical degrees and include the following majors: clinical medicine, preventive medicine (public health), dentistry (stomatology), forensic medicine, medical laboratories, nursing, and pharmacy [14–17]. At medical schools in mainland China, the anatomy syllabus is not exactly the same for medical students of different majors. The teaching contents of human anatomy are designed according to the features of the major. It is noteworthy that not every medical school in mainland China offers all of these majors; usually, clinical medicine, stomatology, and public health have long been established at medical schools. Some medical schools in mainland China have majors in traditional Chinese medicine and nursing (it is different from the coursework offered by nursing schools because students in the former are enrolled in the same clinical medicine courses as undergraduate medical students). Some new majors have also been established at medical schools, such as forensic medicine, medical laboratories, medical imaging and pharmacies, to meet the needs of medical developments. Among these majors, the undergraduate curricula of the basic sciences are quite similar across medical schools in mainland China, and curriculum differences among them mainly exist in clinical curriculum arrangements [18,19]. Anatomy is an essential course for all students in the aforementioned different majors. However, students from clinical medicine study anatomy independent of those in other majors at most medical schools because they are the vast majority of medical undergraduates, whereas anatomy teachers might separately deliver theoretical and practical sessions to students from stomatology, public health or other majors.

In recent years, technological progress has greatly impacted the anatomical learning of medical students [20,21]. These impacts include teaching methods of theoretical and practical sessions, interaction between teaching academics and students and students’ perceptions [22]. Moreover, anatomy education benefits from the flourishing digitalization of human specimens through three-dimensional reorganization technology and virtual technologies [23–25]. The integration of these anatomy teaching resources with online teaching platforms has led to the implementation of anatomical online teaching [26]. To evaluate whether any differences exist in online anatomy teaching between clinical medicine and nonclinical medicine majors, we conducted a nationwide survey among Chinese professional anatomists. Another parallel survey, which focused only on the online anatomy education of clinical medicine students, was simultaneously implemented. The combination of the different viewpoints drawn from complementary or contrasting questions obviously contributes to the anatomy education of students in different majors.

## Methods

### Survey implementation

The survey instrument was designed to collect information about the online teaching of gross anatomy for clinical medicine and nonclinical medicine majors (for example, stomatology, public health, and traditional Chinese medicine) during the COVID-19 pandemic, which ranged from mid-February 2020 to mid-April 2020 in China, including aspects of online anatomy teaching, such as theoretical sessions (lectures), practical sessions, active learning activities, assessments and perceptions of this experience. This grouping of teaching for clinical and nonclinical medicine majors was principally based on the different contact hours for anatomy, generally approximately 100 for clinical medicine majors per semester and 80 or less for nonclinical medicine majors at medical schools in mainland China. Therefore, anatomy is always taught separately to clinical and nonclinical medicine students (stomatology, public health, and traditional Chinese medicine) at medical schools in mainland China. The survey instrument was developed in Chinese (see Supplementary Material 1 for the translated English version). The survey was piloted with the faculty members of the second author to ensure the clarity of the questionnaire and was subsequently revised on the basis of their feedback.

Convenience sampling of the anatomy departments was used. There were 366 anatomical teachers can be reached in a messaging group – the WeChat application (Tencent Holdings Ltd., Shenzhen, China), a popular social media mobile application. These 366 teachers came from the anatomy departments of different medical schools in China. An invitation to participate by completing the survey instrument on an online survey platform, SoJump (Shanghai Information Technology Co., Shanghai, China), was sent to the group members by WeChat. Participation in the survey was voluntary. The survey was performed in collaboration with the Chinese Society for Anatomy Sciences (CSAS), the national organization of anatomical educators. The study was conducted with ethics approval from the Research Ethics Committee of Jinan University (No. MJNER202004006). The names of the surveyed medical schools were de-identified when analysing the data.

### Data analysis

All statistical and graphical analyses were performed using the SPSS statistical package, version 17.0 (IBM Corp., Armonk, NY). The data obtained from the questionnaires were analysed using Kendall’s tau *b* test to assess the correlations of aspects of theoretical and practical online teaching for different majors. The chi-square test was used to assess the statistically significant differences in the data acquired from clinical medicine or nonclinical medicine majors towards gross anatomy education (statistical significance was considered when *P*< 0.05).

## Results

**Figure 1.**
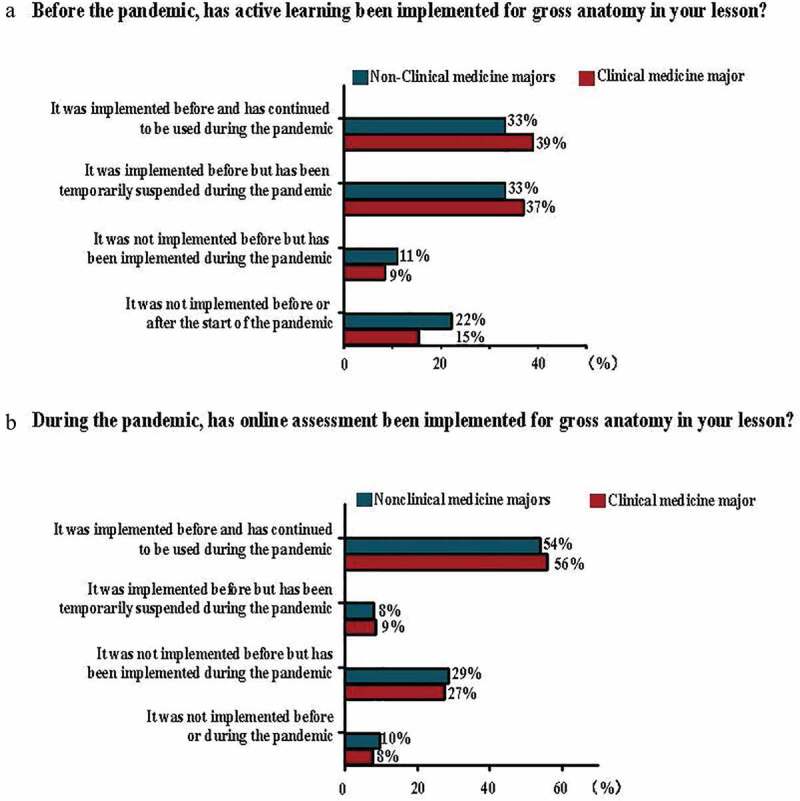
Composition of respondents regarding the majors taught by anatomists and the number of students taught during the pandemic

In this survey, a total of 359 anatomy teachers completed the online questionnaire. After excluding the respondents from Hong Kong and Macau special administrative regions at where the medical education system may differ from that in mainland China, the other 356 answers were included in our analysis of online teaching. The respondents were shown to come from 91 medical schools, covering all the provinces in mainland China. Based on information from the Chinese Ministry of Education (MOE) website, there are currently 140 medical schools in mainland China [27]. Therefore, the results from the survey were representative of the overall situation of anatomy education online. The survey is designed to function using a cross-sectional study method, that is, the anatomy teachers answered the questionnaire based on the medical students they taught in clinical medicine (293) and nonclinical medicine majors (21 from preventive medicine, 13 from stomatology and 29 from traditional Chinese medicine) when the survey was implemented (Figure 1a).

**a**: Pie chart showing the percentages of anatomists who were teaching anatomy courses in clinical or nonclinical medicine majors. **b**: Bar chart showing the percentage of students per class in clinical or nonclinical medicine majors during the COVID-19 pandemic.

### Theoretical and practical sessions

The survey showed that the number of students per class was less than 100 in 57% of the surveyed nonclinical medicine majors (n = 36), whereas more than 301 students per class were taught in 36% of clinical medicine majors (n = 105) A statistically significant difference existed between them (*P*< 0.01) (Figure 1b). Regarding comparisons of online theoretical and practical sessions between nonclinical medicine and clinical medicine majors, we did not find any statistically significant differences between the two groups, including the following items.

**Figure 2.**
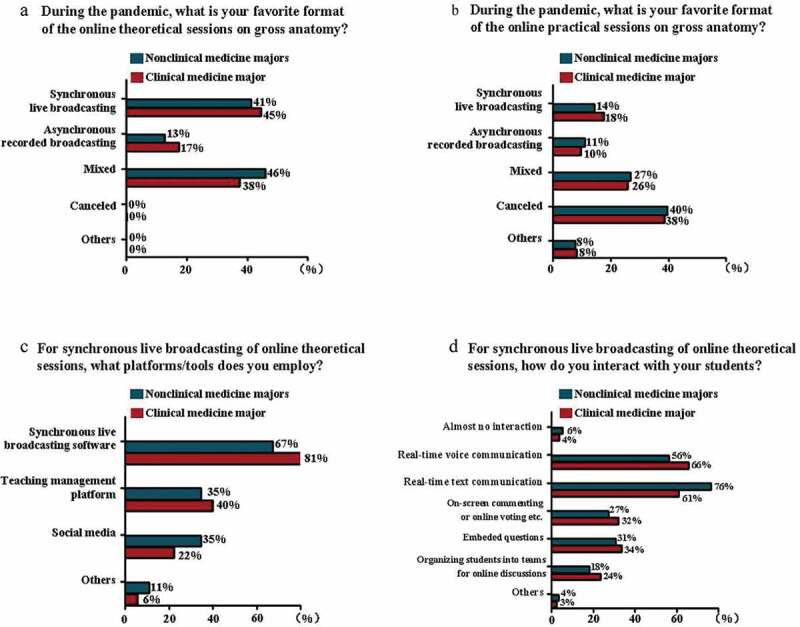
Survey of various online teaching formats of gross anatomy conducted before the pandemic in different majors

**Figure 3.**
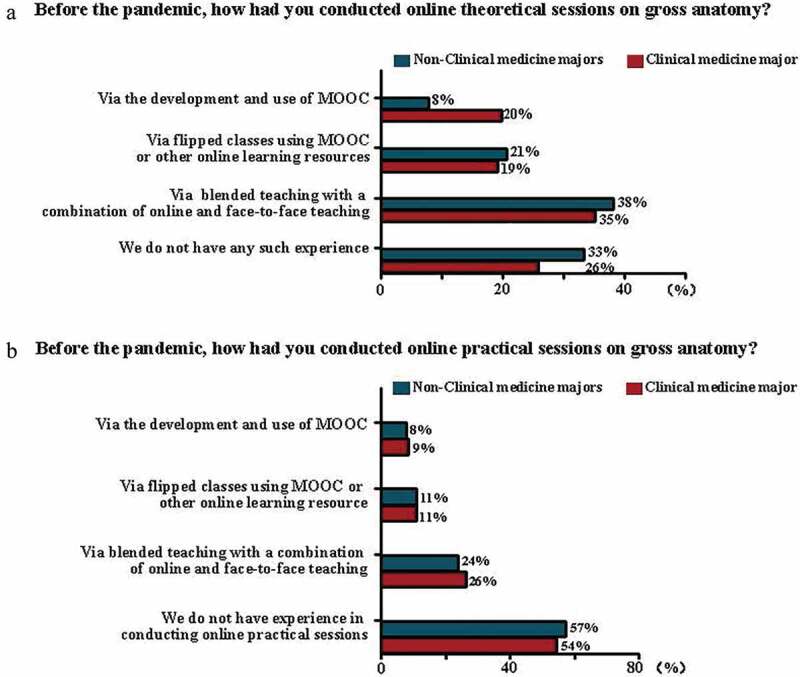
Survey of various aspects regarding gross anatomy online teaching in different majors during the pandemic

Before the pandemic, various online formats of anatomical theoretical sessions were implemented in the classes for nonclinical and clinical medicine majors (n = 42, 67% and n = 219, 74%, respectively) (Figure 2a). During the pandemic, the main formats of online theoretical sessions used for both majors were synchronous live broadcasting (n = 26, 41% and n = 131, 45%, respectively) and mixed synchronous and asynchronous live broadcasting (n = 29, 46% and n = 110, 38%, respectively, Figure 3a); gross anatomy class hours remained unchanged in most nonclinical and clinical medicine majors (n = 49, 78% and n = 211, 72%, respectively); synchronous live broadcasting was adopted in theoretical online anatomy courses in 67% of nonclinical medicine (n = 37) and 81% (n = 195) of clinical medicine majors (Figure 3c). For synchronous live broadcasting of online theoretical sessions, real-time voice (n = 31, 56% and n = 158, 66%, respectively) and text (n = 42, 76% and n = 147, 61%, respectively) communications were the two popular interaction formats with students in nonclinical and clinical medicine majors (Figure 3d), and the majority of the anatomy teachers used less than half of the lecture hours interacting with students in both majors (n = 48, 87% and n = 194, 81%, respectively). For asynchronous live broadcasting of online theoretical sessions, the presentation of a recording screen displaying PowerPoint slides without the appearance of a teacher was mostly used in both majors (n = 24, 65% and n = 117, 73%, respectively). For asynchronous live broadcasting of online theoretical sessions, a teaching management system was mostly used to upload and deliver the materials for both majors (n = 23, 62% and n = 107, 66%, respectively). For either synchronous or asynchronous broadcasting of online theoretical sessions, the teaching materials were adjusted slightly in 43% (n = 27) of nonclinical and 42% (n = 122) of clinical medicine majors.

**a**: Bar chart showing the percentage of various online teaching formats of theoretical gross anatomy sessions conducted in clinical or nonclinical medicine majors before the COVID-19 pandemic. **b**: Bar chart showing percentage of various online teaching formats of theoretical gross anatomy sessions conducted in clinical or nonclinical medicine majors before the COVID-19 pandemic.

**a**: Bar chart showing formats of theoretical gross anatomy online teaching sessions in different majors during the pandemic. **b**: Bar chart showingformats of practical sessions of gross anatomy online teaching in different majors during the pandemic. **c**: Bar chart showing platforms/tools employed for asynchronous live broadcasting of online theoretical sessions of gross anatomy in different majors during the pandemic. **d**: Bar chart showing formats of interactivities for theoretical sessions of gross anatomy online teaching in different majors during the pandemic.

For practical anatomical sessions, 57% (n = 36) of nonclinical and 54% (n = 159) of clinical medicine majors did not experienced online teaching before the pandemic (Figure 2b). During the pandemic, the most commonly used teaching material for practical gross anatomy sessions was anatomical specimens and models in nonclinical and clinical medicine majors (n = 39, 62% and n = 169, 58%, respectively). Practical anatomical sessions were suspended in 40% (n = 25) of nonclinical medicine and 38% (n = 113) of clinical medicine majors (Figure 3b). Having no change in class hours was the most-chosen option for both majors (n = 31, 81% and n = 114, 63%, respectively); 53% (n = 20) of nonclinical medicine and 54% (n = 97) of clinical medicine majors chose to increase the use of anatomical pictures and videos in their practical sessions; 55% (n = 21) of nonclinical medicine and 48% (n = 87) of clinical medicine majors responded that no virtual simulation experiment platform was used; 71% (n = 27) of nonclinical medicine and 71% (n = 127) of clinical medicine majors used less than half of their class hours for teacher-student interactions if practical online sessions were implemented.

### Online active learning and assessments

**Figure 4.**
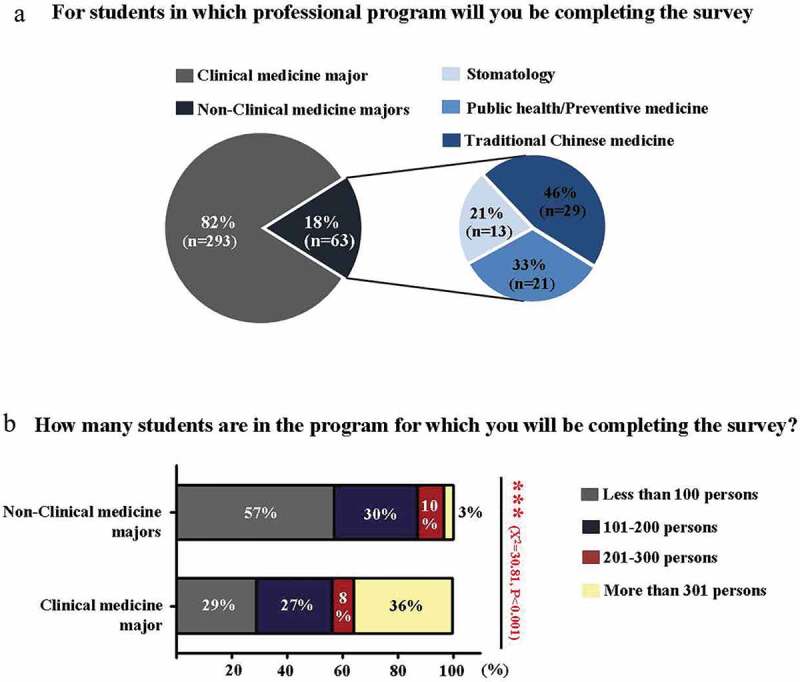
Survey of various aspects regarding active learning and online assessments in gross anatomy in different majors before and during the pandemic

The survey showed no significant differences between nonclinical medicine and clinical medicine majors in implementing active learning in online anatomy courses (n = 42, 66% and n = 223, 76%, respectively, Figure 4a). In detail, most nonclinical and clinical medicine majors implemented different active learning formats, such as flipped classrooms, group discussions, problem-based learning (PBL) and team-based learning (TBL) before the pandemic. For the implementation of active learning during the pandemic, no significant differences exist between nonclinical and clinical medicine majors regarding the increase in class hours for gross anatomy active learning sessions (n = 16, 57% and n = 90, 65%, respectively) and the interactive formats of real-time voice (n = 9, 32% and n = 43, 31%, respectively) or text (n = 7, 25% and n = 53, 38%, respectively) communications.

**a**: Bar chart showing various situations of implementing active learning in gross anatomy in different majors before and during the pandemic. **b**: Bar chart showing various situations of implementing online assessment in gross anatomy in different majors before and during the pandemic.

Regarding the assessment of gross anatomy online courses, the survey suggested no statistically significant difference between nonclinical and clinical medicine majors. First, before and during the pandemic, the assessments were implemented in most anatomy teaching in nonclinical and clinical medicine majors (n = 34, 54% and n = 164, 56%, respectively) (Figure 4b), mainly through the formats of online tests (n = 45, 87% and n = 206, 84%, respectively) and recording class attendance (n = 33, 63% and n = 161, 66%, respectively). Nevertheless, more than half of them (n = 26, 50% and n = 156, 64%, respectively) did not implement the practical component of online tests, instead preferring to display pictures and videos of anatomical specimens and models if implementing practical online tests.

### Evaluations of online teaching during the pandemic

**Figure 5.**
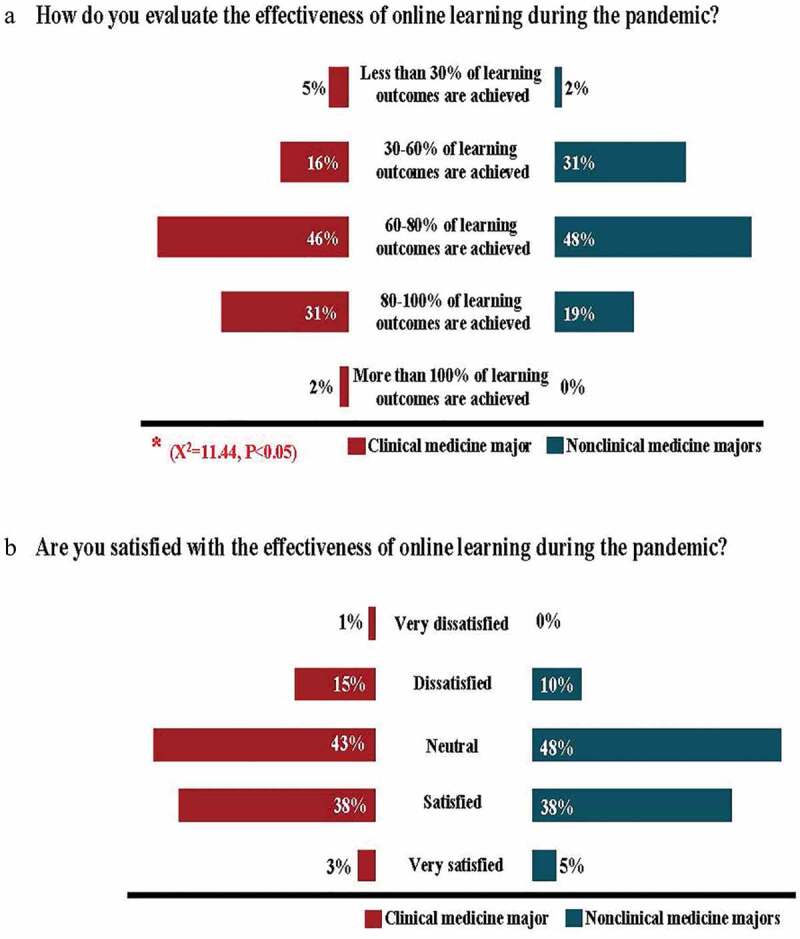
Survey of various aspects regarding the evaluation of effectiveness of online gross anatomy education in different majors during the pandemic

Regarding the evaluation of the effectiveness of online learning, the survey revealed a statistically significant difference between nonclinical and clinical medicine majors (*x^2^ *= 11.44, *P*= 0.02); for example, 48% (n = 30) of the teachers in nonclinical and 46% (n = 135) of the teachers in clinical medicine majors considered that 60–80% of the learning outcomes had been achieved (Figure 5a); 38% of the teachers for both nonclinical (n = 24) and clinical medicine (n = 110) majors were satisfied with online learning during the pandemic (Figure 5b). Teachers from both majors believed that the greatest gains from online learning were a good opportunity to develop novel teaching methods (n = 28, 44% and n = 210, 72%, respectively), a diversity of teaching methods (n = 47, 75% and n = 234, 80%, respectively), and content materials for teaching (n = 45, 71% and n = 222, 76%, respectively). The greatest difficulties mentioned included unstable online teaching environments (n = 31, 49% and n = 170, 58%, respectively), difficulty grasping student progress and learning results (n = 39, 62% and n = 170, 58%, respectively), and an increase in teachers’ workload (n = 24, 38% and n = 75, 26%). In addition, 32% (n = 20) of the nonclinical teachers and 24% (n = 69) of the clinical medicine majors expressed that they would such as to return to traditional face-to-face classes after the pandemic, although similar populations said that they would like to partially implement online teaching.

**a**: Bar chart showing percentages of giving such evaluation indexes on gross anatomy online teaching in different majors during the pandemic. **b**: Bar chart showing satisfaction of gross anatomy online teaching implementation in different majors during the pandemic.

## Discussion

Medical education plays a critical role in any society, especially when the unprecedented COVID-19 pandemic caused disaster worldwide. Adaptive learning and the dynamic shift from traditional face-to-face teaching to online education were the themes in every professional field in 2020. Anatomy is the oldest discipline of medicine; therefore, how anatomy education continued and how it performed online requires more inspection to provide perspectives for assessing the future curriculum design and delivery of anatomical science and, thereafter, to turn the COVID-19 crisis into an opportunity to improve anatomy teaching. Online teaching was derived from distance education, which is defined as implementing educational practice separated by distance and/or time [28]. Today, online teaching and distance education have become blurred because the majority of the distance education providers have made their learning materials fully accessible online to learners via the Internet-based infrastructure of the virtual learning environment. Teaching and the interaction between teachers and students during education have been fulfilled online via synchronous or asynchronous communications and other messaging systems such as email, video, audio, and live text [29]. In the past few decades, given the rapid economic development in China, the central and local governments and private facilitators of online education have provided various products that support the implementation of online teaching, such as online learning materials, online teaching and assessments of students’ learning outcomes, among others. University educators are able to continue moving from traditional face-to-face lectures to online learning in response to the COVID-19 pandemic because of developments in new technologies and improvements in the education infrastructure.

One of the characteristics of nonclinical medicine majors in medical schools in China is that student numbers are much lower than those in clinical medicine majors in the same grade. This difference was reflected in the results in Figure 1; for example, only 6% (n = 21) of the respondents were teaching students from public health or preventive medicine. This problem in the overall Chinese medical education system has received attention during the COVID-19 pandemic, which revealed a shortage of professionals in public health and preventive medicine majors. Nevertheless, the benefit of a small number of students is that anatomy teachers teach students in small classes. Education practitioners have verified that a ‘small-class advantage’ exists when student numbers are properly reduced [30]. Regarding online theoretical and practical anatomy education sessions, no statistically significant differences exist between nonclinical and clinical medicine majors, although the respondents from these majors answered somewhat differently, as revealed by this nationwide survey. For example, before the pandemic, online theoretical anatomy education was widely implemented in teaching for both nonclinical medicine and clinical medicine majors but was less popular among the former. Nevertheless, practical online gross anatomy sessions were not yet popularized in more than half of both majors (Figure 2). A plausible reason for this phenomenon is the ‘hands-on’ nature of the anatomical course. The 3D relationship of the neighbouring structures that are necessary to learn anatomy is a challenge faced by online educators and learners. During the pandemic, teachers in online anatomy classes of both majors predominantly adopted several similar response measures (Figure 3), such as the favourite format of delivering online anatomy sessions (e.g., synchronous live broadcasting), the teaching materials employed (e.g., anatomical specimens and models), the methods used for teacher-student interactions during synchronous live broadcasting of online sessions (e.g., real-time voice or text communications), and the changes in theoretical and practical anatomy class hours. From the standpoint of anatomical science in this survey, we observe that anatomists must prepare specific anatomy teaching resources for online courses before implementing online teaching in comparison with traditional face-to-face teaching [31]. For this reason, many anatomists lacked online teaching experiences in anatomy education before the pandemic, which had nothing to do with the majority of the students who studied human anatomy courses. Therefore, the teaching infrastructure and anatomists’ online teaching experience are especially important to obtain good online teaching results.

Active learning pedagogy has deeply converged in anatomy education worldwide in the past several decades [32,33]. Similarly, the pilot curricula of active learning activities, including flipped classrooms, group discussions, PBL, TBL, and individualized tutoring, have increasingly been employed in anatomy education at medical schools in China [34,35]. This survey showed that active learning activities were implemented in anatomy education in nonclinical and clinical medicine majors before the pandemic (Figure 4). Whether online teaching is successful largely depends on several interpretive factors, including active learning, teacher-student interaction, timing of tasks, and cooperation among students [36]. Active learning is well recognized as being correlated with higher student engagement during online courses that, in turn contributes to effective online learning. Meanwhile, student engagement is more important in the online learning environment than in traditional face-to-face education [37]. Establishing an effective online assessment system in a real-world context is a prerequisite for improving active learning online [38]. This survey demonstrated that an online assessment was implemented before the pandemic and continued to be used during the pandemic in online anatomy courses of both majors without any significant difference between them, including the main formats of online assessment for anatomy education (Figure 4). Teaching online is different; however, as a fundamental component of both teaching and learning, assessment frames students’ achievements and functions as the most effective feedback of what students have learned.

However, we observed a statistically significant difference when evaluating the effectiveness of online learning during the pandemic between nonclinical and clinical medicine majors, with teachers reporting lower effectiveness ratings for anatomical online courses for the nonclinical medicine major, although most of their courses were delivered in small classes (Figure 5). Regarding the greatest gains and difficulties, teachers who were responsible for anatomy sessions for both majors believed that online teaching during the pandemic provided a good chance to develop pedagogically innovative solutions to facilitate learning. They simultaneously reported many difficulties, such as unstable online teaching environments, difficulty grasping student learning outcomes, and a dramatically increased teacher workload. Compared with teachers for clinical medicine majors, teachers who were teaching nonclinical medicine majors were more eager to return to traditional face-to-face teaching after the pandemic.

## Limitations

Admittedly, there are strengths but also several limitations to this study. First, as a cross-sectional study, we did not consider any change over time in every aspect of gross anatomy online teaching (e.g., whether teachers and students gradually adapted to online learning over time) during the pandemic. We principally captured a snapshot of respondents for online teaching of gross anatomy in China in response to the pandemic. Therefore, the findings might not well reflect the longitudinal anatomy educational scene. Second, this comparative research on online teaching in anatomy among different majors was only carried out in the majors of clinical medicine, dentistry and preventive medicine because these three majors enroll the majority of the students at most medical schools in China. Third, the data in this survey from nonclinical medicine were not analysed into further classifications because of the small sample size when compared with those from the clinical medicine major. Fourth, given very few questions in the survey on respondents’ geographic characteristics further analysing the teacher populations who taught anatomy for the two different major types was difficult. Moreover, the evaluation of effectiveness was self-reported by respondents without a clear definition in the survey of effectiveness, which might have indirectly affected the results because students may have different criteria.

## Conclusion

This survey was designed to compare two classes (i.e., nonclinical and clinical medicine majors) of stopgap online education in anatomy at medical schools in China. Generally, the responses from both groups were quite similar regarding the exposed strengths and weaknesses, including aspects of online anatomy theoretical and practical sessions, active learning, assessment and evaluation. Without a doubt, we could foster strengths and circumvent weaknesses only when we are able to identify the obstacles to moving forward on this online education path.

## Supplementary Material

Supplemental Material
