# Supplementary material for: The perceptions of anatomy teachers for different majors during the COVID-19 pandemic: a national Chinese survey
Source: Med Educ Online. 2021 Mar 15;26(1):1897267. doi: 10.1080/10872981.2021.1897267 (PMC7971280; doi:10.1080/10872981.2021.1897267)
Supplement: Supplemental Material [file ZMEO_A_1897267_SM8628.docx]

**Questionnaire on the online teaching of gross anatomy during the COVID-19 pandemic**

We sincerely thank you for participating in this national survey on the online teaching of gross anatomy during the COVID-19 pandemic. The results will provide a summary of our current efforts in organizing online teaching of gross anatomy, identify problems we are facing, and even help to improve the design and quality of online teaching. The questionnaire contains 35 questions, and takes about 5 minutes to complete. There are no standard answers for these questions. Please answer them based on your experience of online teaching since February of 2020. We pledge to keep all data anonymous, and personal information will not be disclosed. Thank you very much again for your participation.

1. What is the name of the university where you work? (The answer to this question is for correlation analyses only, and no personal information will be disclosed) * [Please fill in the space below]

_________________________________

2. For students in which professional program will you be completing the survey? [single choice] *

| ○ Clinical medicine |
| --- |
| ○ Public health/Preventive medicine  ○ Stomatology  ○ Traditional Chinese medicine |

3. How many students are in the program for which you will be completing the survey? [single choice] *

| ○ Less than 100 persons |
| --- |
| ○ 101-200 persons  ○ 201-300 persons  ○ More than 301 persons  **I Gross anatomy theoretical sessions**  4. Before the pandemic, how had your school conducted online theoretical sessions (lectures) on gross anatomy? [single choice] *   \| ○ Via the development and use of MOOC \| \| --- \| \| ○ Via flipped classes using MOOC or other online learning resources \| \| ○ Via blended teaching with a combination of online and face-to-face teaching \| \| ○ We do not have any such experience \| |

5. During the pandemic, what is your favorite format of the online theoretical sessions on gross anatomy? [single choice] *

| ○ Synchronous live broadcasting (SLB): teacher delivers the sessions live (teacher and students engage synchronously) |
| --- |
| ○ Asynchronous recorded broadcasting ARB): teacher records the sessions, and then uploads it to an Internet platform or disperses it to students (teacher and students engage asynchronously). |
| ○ Mixed (some sessions by synchronous and some by asynchronous methods)  ○ Theoretical sessions suspended and will make up the missed lesson after the pandemic (Please go to question 13 directly) |
| ○ Others (please elaborate) _________________ |

6. How have the class hours of theoretical sessions changed in your lesson during the pandemic? [single choice] *

| ○ Increased by more than 50%. |
| --- |
| ○ Increased by 25%-50%. |
| ○ Increased by less than 25% |
| ○ No change |
| ○ Decreased by less than 25%. |
| ○ Decreased by 25%-50% |

○ Decreased by more than 50%

7. For synchronous live broadcasting of online theoretical sessions, what platforms/tools does you employ? (Please go to question 10 if your school does not use synchronous live broadcasting) [Can choose more than one]

| □ Synchronous live broadcasting (SLB) software, such as Tencent Classroom, DingDing, Tencent Meeting, and ZOOM etc. |
| --- |
| □ Teaching management platform with synchronous live broadcasting capability, such as Xuexitong, and Rain Classroom etc. |
| □ Social media, such as QQ, WeChat etc. |
| □ Others (please elaborate) _________________ |

8. For synchronous live broadcasting of online theoretical sessions, how do you interact with your students? [Can choose more than one]

| □ Almost no interaction between teacher and students |
| --- |
| □ Real-time voice communication (on platforms like Tencent Classroom, Tencent Meeting, etc.) |
| □ Real-time text communication (on platforms like WeChat, QQ, etc) |
| □ Real-time on-screen commenting (danmu), online voting etc. |
| □ Embedding questions in PowerPoint presentations |
| □ Organizing students into teams for online discussions |
| □ Others (please elaborate) _________________ |

9. For synchronous live broadcasting of online theoretical sessions, how much time do you use for student-teacher interaction? [single choice]

| ○ More than half of the lecture time |
| --- |
| ○ Less than half of the lecture time |
| ○ Almost no interaction between teacher and students. The teacher lectures most of the time. |

10. For asynchronous recorded broadcasting of online theoretical sessions, how do you record the sessions? (If you do not use asynchronous recorded broadcasting, please go to question 12 directly) [single choice]

| ○ Use a video camera to record the teacher delivering the session, without post-production |
| --- |
| ○ Use a video camera to record the teacher delivering the session, with post-production |
| ○ Use a screen capture software to record only the slides and sound of the PowerPoint presentation, and the teacher does not appear in the recorded session |
| ○ Others (please elaborate) _________________ |

11. For asynchronous recorded broadcasting of online theoretical sessions, what platforms/tools do you employ for delivering the recorded session? [Can choose more than one]

| □ Platforms for online courses, such as Chinese University MOOC, People's Medical Publishing House MOOC, and Zhihuishu etc. |
| --- |
| □ Teaching management systems, such as Xuexitong, and Rain Classroom, BB (blackboard) Internet Teaching Platform, etc. |
| □ Social media, such as QQ, WeChat, etc. |
| □ Others (please elaborate) _________________ |

12. For either synchronous or asynchronous broadcasting of online theoretical sessions, what changes were made to the content materials? [single choice]

| ○ The content materials are the same as those for face-to-face teaching, and have not been changed |
| --- |
| ○ The content materials have been adjusted slightly  ○ The content materials have been broken down into smaller parts |
| ○ The content materials have been completely redesigned |
| **II Gross anatomy practical sessions** |

13. Before the pandemic, how had you conducted online practical sessions on gross anatomy? [single choice] *

| ○ Via the development and use of MOOC |  |  |
| --- | --- | --- |
| ○ Via flipped classes using MOOC or other online learning resources |  |  |
| ○ Via blended teaching with a combination of online and face-to-face teaching |  |  |
| ○ We do not have experience in conducting online practical sessions |  |  |

14. What kinds of teaching materials were used in your practical sessions before the pandemic? [Can choose more than one] *

| ○ Digital human anatomy software |
| --- |
| ○ Pictures and videos (including anatomy dissection video) |
| ○ Dissection |
| ○ Specimens and models |

15. During the pandemic, what is your favorite format of the online practical sessions on gross anatomy? [single choice] *

| ○ Synchronous live broadcasting (SLB): teacher delivers the sessions live (teacher and students engage synchronously) |  |
| --- | --- |
| ○ Asynchronous recorded broadcasting (ARB): teacher records the sessions, and then uploads it to an internet platform or disperses it to students (teacher and students engage asynchronously). |  |
| ○ Mixed (some sessions by synchronous and some by asynchronous methods)  ○ Practical sessions are suspended and will make-up sessions will be implemented when face-to-face classes resume (Please go to question 20 directly) |  |
| ○ Others (please elaborate) _________________ |  |
|  |  |

16. During the pandemic, how have the class hours of gross anatomy practical sessions changed in your lesson? [single choice] *

| ○ Increased by more than 50% |
| --- |

○ Increased by 25%-50%

○ Increased by less than 25%

○ No change

○ Decreased by less than 25%

○ Decreased by 25%-50%

○ Decreased by more than 50%

17. During the pandemic, how has the teaching materials for online practical sessions changed in your lesson? [Can choose more than one] *

○ No change

| ○ Increased use of digital human anatomy software by less than 50%  ○ Increased use of digital human anatomy software by more than 50% |
| --- |
| ○ Increased use of pictures and videos (including dissection videos) by less than 50% |
| ○ Increased use of pictures and videos (including dissection videos) by more than 50% |

18. During the pandemic, what platforms/tools do you employ for gross anatomy practical sessions? [single choice] *

| ○ The National Virtual Simulation Experiment Teaching Center (the modules relevant to anatomy) |
| --- |
| ○ The Provincial Virtual Simulation Experiment Teaching Center |
| ○ The virtual simulation experiment platform built by your school |

○ No virtual simulation experiment platform is used

19. How much time do you use for teacher-student interaction during the online anatomical practical sessions? [single choice]

| ○ More than half of the class time |
| --- |
| ○ Less than half of the class time |
| ○ Almost no interaction between teacher and students. The teacher talks most of the time. |

20. During the pandemic, what are the changes in body donation in your schools? [single choice] *

○ Your school does not have a body donation program

| ○ Body donation is temporarily suspended during the pandemic  ○ Your school received more donated bodies than before |
| --- |
| ○ Your school received similar number of donated bodies as before |
| ○ Your school received fewer donated bodies than before |

**III Active learning sessions**

21. Before the pandemic, has active learning (e.g., flipped classroom, group discussion, problem-based learning, team-based learning, and individualized tutoring) been implemented for gross anatomy in your lesson? [single choice] *

| ○ It was implemented before and has continued to be used during the pandemic |
| --- |
| ○ It was implemented before but has been temporarily suspended during the pandemic (please go to question 25 directly) |
| ○ It was not implemented before but has been implemented during the pandemic |
| ○ It was not implemented before or after the start of the pandemic (please go to question 25 directly) |

22. During the pandemic, what types of active learning have been implemented for gross anatomy in your lesson? [Can choose more than one]

| □ Flipped classroom |
| --- |
| □ Group discussion |
| □ PBL (Problem-based learning) |
| □ TBL (Team-based learning)  □ Individualized tutoring  □ Others (please elaborate) _________________ |

23. During the pandemic, how have the class hours of gross anatomy active learning sessions changed in your lesson? [single choice] *

| ○ Increased by more than 50% |
| --- |

○ Increased by 25%-50%

○ Increased by less than 25%

○ No change

○ Decreased by less than 25%

○ Decreased by 25%-50%

○ Decreased by more than 50%

24. During the pandemic, what tool is employed to implement active learning of gross anatomy in your lesson? [single choice]

| ○ Real-time voice communication (on platforms like Tencent Classroom, Tencent Meeting, etc.)  ○ Real-time text communication (on platforms like WeChat, QQ, etc) |
| --- |
| ○ Real-time on-screen commenting (danmu), online voting etc.  ○ Embedding questions in PowerPoint presentations |
| ○ Platforms for online courses (e.g., Blackboard) |
| ○ Others (please elaborate) _________________  **IV Assessment** |

25. Before and during the pandemic, has online assessment been implemented for gross anatomy in your lesson? [single choice] *

| \| ○ It was implemented before and has continued to be used during the pandemic \| \| --- \| \| ○ It was implemented before but has been temporarily suspended during the pandemic (please go to question 28 directly) \| \| ○ It was not implemented before but has been implemented during the pandemic \| \| ○ It was not implemented before or during the pandemic (please go to question 28 directly) \| |
| --- | --- | --- | --- | --- |

| 26. During the pandemic, what are the main formats of online assessment for gross anatomy in your lesson? [Can choose more than one]   \| □ Online tests \| \| --- \| \| □ Peer assessment of homework \| \| □ Recording the attendance for online sessions \| \| □ Subjective assessment by teachers \| \| □ Others (please elaborate) _________________ \| |
| --- | --- | --- | --- | --- | --- |
|  |
| 27. During the pandemic, how do you implement the practical aspect of online test? [Can choose more than one]   \| □ The online tests do not have practical component \| \| --- \| \| □ Via pictures and videos of specimens and models \| \| □ Via digital human anatomy software \| \| □ Others (please elaborate) _________________ \| |
|  |
| **V Evaluation and other aspects** |
| 28. How do you evaluate the effectiveness of online learning during the pandemic? [single choice] *   \| ○ Less than 30% of learning outcomes are achieved \| \| --- \| \| ○ 30-60% of learning outcomes are achieved \| \| ○ 60-80% of learning outcomes are achieved \| \| ○ 80-100% of learning outcomes are achieved \| \| ○ More than 100% of learning outcomes are achieved \| |
|  |

29. Are you satisfied with the effectiveness of online learning during the pandemic? [single choice] *

| ○ Very satisfied |
| --- |
| ○ Satisfied |
| ○ Neutral |
| ○ Dissatisfied |
| ○ Very dissatisfied |

30. What are your biggest gains from the online teaching during the pandemic? [Can choose up to 3] *

| □ Good opportunity for developing novel teaching methods |
| --- |
| □ Diversity of teaching methods |
| □ Development of content materials for teaching |
| □ Enhancement of interaction between teachers and students |
| □ Expediting the feedback process for students |
| □ Development of more individualized instruction |
| □ Others (please elaborate) _________________ |

31. What are the biggest difficulties you have encountered in implementing online teaching? [Can choose up to 3] *

| □ Insufficient training and management for online teaching from school |
| --- |
| □ Unstable online teaching environments, platforms and tools |
| □ Unfamiliarity with online teaching technique, platforms and tools |
| □ Not adapting to novel teaching concepts and methods |
| □ Insufficient online teaching resources |
| □ Content materials that are not easily amenable to online teaching |
| □ Difficulty in grasping student progress and results of learning |
| □ Difficulty in interacting effectively with students |
| □ The much longer time needed for preparing online teaching than for traditional teaching |
| □ Others (please elaborate) _________________ |

32. Would you like to continue online teaching after the pandemic? [single choice] *

| ○ Willing to continue to conduct the theoretical sessions online |
| --- |
| ○ Willing to continue to conduct practical sessions online |
| ○ Willing to continue to conduct both theoretical and practical sessions online |
| ○ Willing to continue to conduct some theoretical and practical sessions online |
| ○ Prefer returning to traditional face-to-face classes |

33. Your gender? [single choice] *

| ○ Male |
| --- |
| ○ Female |

33. Your age? [single choice] *

| ○ 20-29 years old |
| --- |
| ○ 30-39 years old |
| ○ 40-49 years old |
| ○ 50-59 years old |
| ○ >60 years old |
|  |

35. What suggestions do you have for the sustainable development of online teaching of gross anatomy? [Please fill in the space below]

_________________________________
